# Supplementary material for: High vaccine effectiveness against severe COVID-19 in the elderly in Finland before and after the emergence of Omicron
Source: BMC Infect Dis. 2022 Nov 5;22:816. doi: 10.1186/s12879-022-07814-4 (PMC9636823; doi:10.1186/s12879-022-07814-4)

Supplementary Data

High vaccine effectiveness against severe Covid-19 in the elderly in Finland before and after the emergence of Omicron

Ulrike Baum, Eero Poukka, Tuija Leino, Terhi Kilpi, Hanna Nohynek, Arto Palmu

Table S1: Highly predisposing comorbidities or medical therapies . . . 2
Table S2: Moderately predisposing comorbidities or medical therapies . . . 3
Table S3: Enrollment of the study cohort . . . 4
Table S4: Distribution of baseline characteristics . . . 5
Table S5: Number of days until first dose . . . 6
Table S6: Number of Covid-19-related hospital admissions . . . 7
Table S7: Number of Covid-19-related ICU admissions . . . 8
Table S8: VE against Covid-19-related hospital admission . . . 9
Table S9: VE against Covid-19-related ICU admission . . . 10
Table S10: VE against Covid-19-related hospital admission by age group . . . 11
Table S11: VE against Covid-19-related hospital admission by presence of comorbidities . . . 12
Table S12: VE against Covid-19-related hospital admission in 2021 Q4 . . . 13
Table S13: VE against Covid-19-related hospital admission in 2022 Q1 . . . 14
Table S14: Negative control outcome analysis . . . 15
Figure S1: Distribution of SARS-CoV-2 variants by calendar month . . . 16
Figure S2: Distribution of exposure states by calendar month . . . 17
Figure S3: Number of cases by calendar month . . . 18
Figure S4: Cumulative hazard of Covid-19-related hospital admission . . . 19
Figure S5: Cumulative hazard of negative control outcome . . . 20

Table S1: Highly predisposing comorbidities or medical therapies Definition of comorbidities and medical therapies that were considered highly predisposing to severe Covid-19.

Classification system	Codes
**Actively treated cancer**	
ICD10	C00, C01, C02, C03, C04, C05, C06, C07, C08, C09, C10, C11, C12, C13, C14, C15, C16, C17, C18, C19, C20, C21, C22, C23, C24, C25, C26, C27, C28, C29, C30, C31, C32, C33, C34, C35, C36, C37, C38, C39, C40, C41, C42, C43, C45, C46, C47, C48, C49, C50, C51, C52, C53, C54, C55, C56, C57, C58, C59, C60, C61, C62, C63, C64, C65, C66, C67, C68, C69, C70, C71, C72, C73, C74, C75, C76, C77, C78, C79, C80, C81, C82, C83, C84, C85, C86, C87, C88, C89, C90, C91, C92, C93, C94, C95, C96, C97, D051, D39
**Down syndrome**	
ICD10	Q90
**Organ or stem cell transplantation**	
ICD10	T86, Z94
**Severe chronic respiratory disease**	
ICD10	E84, J41, J42, J43, J44, J45, J46, J47, Z902
ICPC2	R96
**Severe disorder of the immune system**	
ICD10	D7081, D7089, D80, D81, D82, D83, D84, E3100, E723, E76, E791, E792, E798, E799, Q7782, Q7800, Q7808, Q809, Q8900, Q8933, Q8934, Q938, Z908
NCSP	JMA
**Severe kidney disease**	
ICD10	E102, E112, E142, I12, I13, N00, N01, N02, N03, N04, N05, N07, N08, N11, N14, N18, N19
**Type 2 diabetes mellitus**	
ATC	A10B
ICD10	E11, E13, E14
ICPC2	T90

ATC, Anatomical Therapeutic Chemical Classification System;

ICD10, International Statistical Classification of Diseases, tenth revision;

ICPC2, International Classification of Primary Care, second edition;

NCSP, Nordic Classification of Surgical Procedures

Table S2: Moderately predisposing comorbidities or medical therapies Definition of comorbidities and medical therapies that were considered moderately predisposing to severe Covid-19.

Classification system	Codes
**Autoimmune disease**	
ICD10	D86, K50, K51, L40, M02, M05, M06, M07, M139, M45, M460, M461, M469, M941
**Immunosuppressive medication**	
ATC	H02AB02, H02AB04, H02AB06, H02AB07, L01BA01, L01XC02, L04AA06, L04AA10, L04AA13, L04AA18, L04AA24, L04AA26, L04AA29, L04AA33, L04AA37, L04AB, L04AC, L04AD01, L04AD02, L04AX01, L04AX03
**Neurological condition affecting breathing**	
ICD10	G20, G21, G22, G23, G24, G25, G26, G70, G71, G72, G73, G80, G81, G82, G83, I60, I61, I62, I63, I64, I65, I66, I67, I68, I69
**Psychotic disease**	
ATC	N05AH02
ICD10	F20, F21, F22, F23, F24, F25, F26, F27, F28, F29
ICPC2	P72
**Severe chronic liver disease**	
ICD10	K702, K703, K704, K71, K72, K73, K74
**Severe heart disease**	
ICD10	I110, I119, I12, I130, I131, I132, I139, I15, I20, I21, I22, I23, I24, I25, I26, I27, I28, I418, I42, I43, I50
**Sleep apnea**	
ICD10	G473
NCSP	WX723, WX780
**Type 1 diabetes mellitus or adrenal insufficiency**	
ATC	A10A
ICD10	E10, E250, E271, E272, E274, E3100, E3101, E3108, E896
ICPC2	T89

ATC, Anatomical Therapeutic Chemical Classification System;

ICD10, International Statistical Classification of Diseases, tenth revision;

ICPC2, International Classification of Primary Care, second edition;

NCSP, Nordic Classification of Surgical Procedures

Table S3: Enrollment of the study cohort Individuals were excluded if they had experienced any of the listed events prior to the start of the study.

	Number of individuals
Target population	898079
Excluded due to prior laboratory-confirmed SARS-CoV-2 infection	1856
Excluded due to prior Covid-19-related hospital admission	<5
Excluded due to prior Covid-19-related ICU admission	<5
Excluded due to prior Covid-19 vaccination	<5
Study cohort	896220

Table S4: Distribution of baseline characteristics

	n	%
**Age in years**		
70-79	582456	65
80-89	257232	29
90-115	56532	6
**Sex**		
Male	381794	43
Female	514426	57
**Region of residence**		
Helsinki-Uusimaa	215597	24
Åland	4884	1
Northern and Eastern Finland	222845	25
Southern Finland	214011	24
Western Finland	238883	27
**In long-term care**		
No	836441	93
Yes	59779	7
**Influenza vaccination in 2019–2020**		
No	394840	44
Yes	501380	56
**Nights hospitalized between 2015 and 2019**		
0	460923	51
1-5	191252	21
6-20	137503	15
21+	106542	12
**Presence of comorbidities or medical therapies**		
No predisposing comorbidities or medical therapies	283025	32
Only moderately predisposing comorbidities or medical therapies	249467	28
At least one highly predisposing comorbidity or medical therapy	363728	41
**Presence of particular comorbidities or medical therapies**		
Severe heart disease	366099	41
Type 2 diabetes mellitus	197819	22
Immunosuppressive medication	138701	15
Actively treated cancer	124874	14
Severe chronic respiratory disease	101222	11
Neurological condition affecting breathing	89043	10
Autoimmune disease	64897	7
Type 1 diabetes mellitus or adrenal insufficiency	52394	6
Sleep apnea	46101	5
Severe kidney disease	22440	3
Psychotic disease	11357	1
Severe chronic liver disease	4229	0
Organ or stem cell transplantation	2187	0
Severe disorder of the immune system	1555	0
Down syndrome	15	0

Table S5: Number of days until first dose Distribution of the number of days until the receipt of the first dose in the study of vaccine effectiveness against Covid-19-related hospital admission.

	Percentile		
	25th	50th	75th
**Age in years**			
70-79	80	92	102
80-89	50	60	73
90-115	32	52	66
**Sex**			
Male	66	86	100
Female	60	80	94
**Region of residence**			
Helsinki-Uusimaa	59	80	93
Åland	48	69	84
Northern and Eastern Finland	62	82	101
Southern Finland	65	85	100
Western Finland	64	81	95
**In long-term care**			
No	66	84	97
Yes	23	30	47
**Influenza vaccination in 2019–2020**			
No	64	86	102
Yes	61	80	94
**Nights hospitalized between 2015 and 2019**			
0	68	87	101
1-5	65	81	95
6-20	57	74	93
21+	38	61	87
**Presence of comorbidities or medical therapies**			
No predisposing comorbidities or medical therapies	67	87	101
Only moderately predisposing comorbidities or medical therapies	59	80	94
At least one highly predisposing comorbidity or medical therapy	60	80	94

Table S6: Number of Covid-19-related hospital admissions by vaccine, dose and days since last vaccination. The table shows the number of cases and person-years, the cumulative risk (per 100000) at the end of the study estimated based on the Kaplan-Meier estimator, the number of study subjects who contributed person-time, the number of study subjects who were censored before the end of the study and the number of study subjects who were still considered at risk at the end of the study. Study subjects may have passed through multiple exposure states before becoming cases or being censored.

	Cases	P-years	Risk	Subjects	Censored	At risk
Not vaccinated	793	244838	1313	896219	19439	34185
Comirnaty 0-20	50	40761	951	709617	1274	144
Comirnaty 21-83	84	114708	952	694046	6481	729
Comirnaty 84+	34	10142	535	82994	5174	4257
Comirnaty + Comirnaty 0-13	9	26489	442	691558	636	408
Comirnaty + Comirnaty 14-90	33	144412	108	690508	8215	4065
Comirnaty + Comirnaty 91-180	210	158116	277	677291	9830	14194
Comirnaty + Comirnaty 181+	200	31354	457	503537	7918	24383
Comirnaty + Comirnaty + Comirnaty 0-13	40	20968	186	548987	663	2492
Comirnaty + Comirnaty + Comirnaty 14-60	107	69468	84	545832	7024	8223
Comirnaty + Comirnaty + Comirnaty 61+	359	74262	125	530585	48349	482236
Comirnaty + Comirnaty + Spikevax 0-13	10	2787	69	72922	73	279
Comirnaty + Comirnaty + Spikevax 14-60	28	9222	118	72570	723	2907
Comirnaty + Comirnaty + Spikevax 61+	39	5601	325	68940	1674	67266
Spikevax 0-20	<5	4901	386	85310	171	13
Spikevax 21-83	10	13902	466	85094	2470	187
Spikevax 84+	8	1662	825	13902	1917	851
Spikevax + Spikevax 0-13	<5	3053	9	79701	77	62
Spikevax + Spikevax 14-90	5	16589	201	79561	1126	812
Spikevax + Spikevax 91-180	34	17796	277	77410	1319	2308
Spikevax + Spikevax 181+	23	3315	433	53206	784	3232
Spikevax + Spikevax + Comirnaty 0-13	<5	666	175	17504	38	164
Spikevax + Spikevax + Comirnaty 14-60	6	2167	137	17302	374	666
Spikevax + Spikevax + Comirnaty 61+	10	2092	70	16262	1314	14948
Spikevax + Spikevax + Spikevax 0-13	5	2006	70	52477	75	169
Spikevax + Spikevax + Spikevax 14-60	8	6630	27	52233	746	1113
Spikevax + Spikevax + Spikevax 61+	41	5948	109	50374	2585	47789
Vaxzevria 0-20	<5	2740	14	47669	45	0
Vaxzevria 21-83	<5	7965	7	47612	5404	0
Vaxzevria 84+	<5	474	342	5100	2812	172
Vaxzevria + Vaxzevria 0-13	0	1504	0	39236	17	0
Vaxzevria + Vaxzevria 14-90	<5	8248	16	39219	208	0
Vaxzevria + Vaxzevria 91-180	23	9067	205	38962	400	6
Vaxzevria + Vaxzevria 181+	18	1283	379	24663	319	1896
Vaxzevria + Vaxzevria + Comirnaty 0-13	<5	919	49	24014	19	74
Vaxzevria + Vaxzevria + Comirnaty 14-60	<5	3044	24	23921	224	669
Vaxzevria + Vaxzevria + Comirnaty 61+	12	2434	363	23028	468	22560
Vaxzevria + Vaxzevria + Spikevax 0-13	<5	474	31	12376	7	12
Vaxzevria + Vaxzevria + Spikevax 14-60	<5	1573	95	12357	79	536
Vaxzevria + Vaxzevria + Spikevax 61+	5	939	102	11742	111	11631

Table S7: Number of Covid-19-related ICU admissions by vaccine, dose and days since last vaccination. The table shows the number of cases and person-years, the cumulative risk (per 100000) at the end of the study estimated based on the Kaplan-Meier estimator, the number of study subjects who contributed person-time, the number of study subjects who were censored before the end of the study and the number of study subjects who were still considered at risk at the end of the study. Study subjects may have passed through multiple exposure states before becoming cases or being censored.

	Cases	P-years	Risk	Subjects	Censored	At risk
Not vaccinated	159	244856	238	896219	19408	34215
Comirnaty 0-20	10	40762	75	709618	1246	144
Comirnaty 21-83	8	114711	14	694075	6496	729
Comirnaty 84+	<5	10143	68	83006	5184	4259
Comirnaty + Comirnaty 0-13	<5	26489	2	691560	630	408
Comirnaty + Comirnaty 14-90	<5	144413	8	690516	8212	4065
Comirnaty + Comirnaty 91-180	26	158121	29	677301	9788	14199
Comirnaty + Comirnaty 181+	22	31360	55	503584	7954	24393
Comirnaty + Comirnaty + Comirnaty 0-13	<5	20969	2	548989	626	2492
Comirnaty + Comirnaty + Comirnaty 14-60	9	69472	3	545871	7021	8227
Comirnaty + Comirnaty + Comirnaty 61+	26	74271	55	530623	48271	482352
Comirnaty + Comirnaty + Spikevax 0-13	<5	2787	15	72922	65	279
Comirnaty + Comirnaty + Spikevax 14-60	5	9222	13	72578	724	2907
Comirnaty + Comirnaty + Spikevax 61+	<5	5602	4	68947	1671	67276
Spikevax 0-20	0	4901	0	85310	168	13
Spikevax 21-83	<5	13902	4	85097	2473	187
Spikevax 84+	0	1662	0	13902	1915	853
Spikevax + Spikevax 0-13	0	3053	0	79701	76	62
Spikevax + Spikevax 14-90	0	16589	0	79562	1125	812
Spikevax + Spikevax 91-180	<5	17797	45	77412	1317	2308
Spikevax + Spikevax 181+	<5	3316	30	53210	784	3235
Spikevax + Spikevax + Comirnaty 0-13	0	666	0	17504	35	164
Spikevax + Spikevax + Comirnaty 14-60	0	2167	0	17305	376	666
Spikevax + Spikevax + Comirnaty 61+	0	2092	0	16263	1309	14954
Spikevax + Spikevax + Spikevax 0-13	0	2007	0	52478	70	170
Spikevax + Spikevax + Spikevax 14-60	0	6630	0	52238	749	1113
Spikevax + Spikevax + Spikevax 61+	0	5949	0	50376	2573	47803
Vaxzevria 0-20	0	2740	0	47669	42	0
Vaxzevria 21-83	<5	7965	4167	47615	5408	0
Vaxzevria 84+	0	474	0	5099	2811	172
Vaxzevria + Vaxzevria 0-13	0	1504	0	39236	17	0
Vaxzevria + Vaxzevria 14-90	<5	8248	10	39219	208	0
Vaxzevria + Vaxzevria 91-180	5	9067	13	38962	397	6
Vaxzevria + Vaxzevria 181+	<5	1283	71	24666	322	1896
Vaxzevria + Vaxzevria + Comirnaty 0-13	0	919	0	24014	17	74
Vaxzevria + Vaxzevria + Comirnaty 14-60	0	3044	0	23923	226	669
Vaxzevria + Vaxzevria + Comirnaty 61+	<5	2434	307	23028	462	22566
Vaxzevria + Vaxzevria + Spikevax 0-13	0	474	0	12376	6	12
Vaxzevria + Vaxzevria + Spikevax 14-60	0	1573	0	12358	77	536
Vaxzevria + Vaxzevria + Spikevax 61+	<5	939	11	11745	111	11634

Table S8: VE against Covid-19-related hospital admission Vaccine effectiveness (in %) quantified as one minus the hazard ratio adjusted for age, sex, region of residence, residence in a long-term care facility, influenza vaccination in 2019–2020, number of nights hospitalized between 2015 and 2019 and presence of predisposing comorbidities or medical therapies.

	MLE	LCI	UCI	p-value[1]
Comirnaty 0-20	45	26	60	.
Comirnaty 21-83	61	49	71	.
Comirnaty 84+	64	49	75	.
Comirnaty + Comirnaty 0-13	80	61	90	.
Comirnaty + Comirnaty 14-90	93	89	95	.
Comirnaty + Comirnaty 91-180	85	82	87	.
Comirnaty + Comirnaty 181+	69	63	74	.
Comirnaty + Comirnaty + Comirnaty 0-13	89	85	92	.
Comirnaty + Comirnaty + Comirnaty 14-60	95	94	96	.
Comirnaty + Comirnaty + Comirnaty 61+	89	87	91	.
Comirnaty + Comirnaty + Spikevax 0-13	87	75	93	.
Comirnaty + Comirnaty + Spikevax 14-60	91	87	94	.
Comirnaty + Comirnaty + Spikevax 61+	85	78	89	.
Spikevax 0-20	72	24	90	.
Spikevax 21-83	70	43	84	.
Spikevax 84+	63	26	82	.
Spikevax + Spikevax 0-13	86	0	98	.
Spikevax + Spikevax 14-90	93	82	97	.
Spikevax + Spikevax 91-180	82	74	87	.
Spikevax + Spikevax 181+	73	59	83	.
Spikevax + Spikevax + Comirnaty 0-13	78	32	93	.
Spikevax + Spikevax + Comirnaty 14-60	91	81	96	.
Spikevax + Spikevax + Comirnaty 61+	90	80	94	.
Spikevax + Spikevax + Spikevax 0-13	90	75	96	.
Spikevax + Spikevax + Spikevax 14-60	97	93	98	.
Spikevax + Spikevax + Spikevax 61+	87	82	91	.
Vaxzevria 0-20	32	-116	78	.
Vaxzevria 21-83	68	-3	90	.
Vaxzevria 84+	37	-95	80	.
Vaxzevria + Vaxzevria 0-13	100	.	.	0.132
Vaxzevria + Vaxzevria 14-90	81	49	93	.
Vaxzevria + Vaxzevria 91-180	74	60	83	.
Vaxzevria + Vaxzevria 181+	48	17	68	.
Vaxzevria + Vaxzevria + Comirnaty 0-13	84	51	95	.
Vaxzevria + Vaxzevria + Comirnaty 14-60	98	91	99	.
Vaxzevria + Vaxzevria + Comirnaty 61+	88	78	93	.
Vaxzevria + Vaxzevria + Spikevax 0-13	92	41	99	.
Vaxzevria + Vaxzevria + Spikevax 14-60	94	82	98	.
Vaxzevria + Vaxzevria + Spikevax 61+	87	70	95	.

MLE, maximum likelihood estimate;

LCI/UCI, lower/upper limit of the 95% Wald confidence interval

[1] Likelihood-ratio test

Table S9: VE against Covid-19-related ICU admission Vaccine effectiveness (in %) quantified as one minus the hazard ratio adjusted for age, sex, region of residence, residence in a long-term care facility, influenza vaccination in 2019–2020, number of nights hospitalized between 2015 and 2019 and presence of predisposing comorbidities or medical therapies.

	MLE	LCI	UCI	p-value[1]
Comirnaty 0-20	57	13	79	.
Comirnaty 21-83	84	63	93	.
Comirnaty 84+	69	15	89	.
Comirnaty + Comirnaty 0-13	75	-10	94	.
Comirnaty + Comirnaty 14-90	98	92	99	.
Comirnaty + Comirnaty 91-180	92	87	95	.
Comirnaty + Comirnaty 181+	79	65	87	.
Comirnaty + Comirnaty + Comirnaty 0-13	99	91	100	.
Comirnaty + Comirnaty + Comirnaty 14-60	98	95	99	.
Comirnaty + Comirnaty + Comirnaty 61+	95	92	97	.
Comirnaty + Comirnaty + Spikevax 0-13	94	54	99	.
Comirnaty + Comirnaty + Spikevax 14-60	92	79	97	.
Comirnaty + Comirnaty + Spikevax 61+	96	84	99	.
Spikevax 0-20	100	.	.	0.016
Spikevax 21-83	70	-29	93	.
Spikevax 84+	100	.	.	0.018
Spikevax + Spikevax 0-13	100	.	.	0.122
Spikevax + Spikevax 14-90	100	.	.	<0.001
Spikevax + Spikevax 91-180	95	80	99	.
Spikevax + Spikevax 181+	93	46	99	.
Spikevax + Spikevax + Comirnaty 0-13	100	.	.	0.022
Spikevax + Spikevax + Comirnaty 14-60	100	.	.	<0.001
Spikevax + Spikevax + Comirnaty 61+	100	.	.	<0.001
Spikevax + Spikevax + Spikevax 0-13	100	.	.	<0.001
Spikevax + Spikevax + Spikevax 14-60	100	.	.	<0.001
Spikevax + Spikevax + Spikevax 61+	100	.	.	<0.001
Vaxzevria 0-20	100	.	.	0.074
Vaxzevria 21-83	68	-151	96	.
Vaxzevria 84+	100	.	.	0.140
Vaxzevria + Vaxzevria 0-13	100	.	.	0.509
Vaxzevria + Vaxzevria 14-90	78	2	95	.
Vaxzevria + Vaxzevria 91-180	81	52	93	.
Vaxzevria + Vaxzevria 181+	64	-14	89	.
Vaxzevria + Vaxzevria + Comirnaty 0-13	100	.	.	0.002
Vaxzevria + Vaxzevria + Comirnaty 14-60	100	.	.	<0.001
Vaxzevria + Vaxzevria + Comirnaty 61+	96	71	99	.
Vaxzevria + Vaxzevria + Spikevax 0-13	100	.	.	0.021
Vaxzevria + Vaxzevria + Spikevax 14-60	100	.	.	<0.001
Vaxzevria + Vaxzevria + Spikevax 61+	89	22	99	.

MLE, maximum likelihood estimate;

LCI/UCI, lower/upper limit of the 95% Wald confidence interval

[1] Likelihood-ratio test

Table S10: VE against Covid-19-related hospital admission by age group Vaccine effectiveness (in %) quantified as one minus the hazard ratio stratified by age in years and adjusted for sex, region of residence, residence in a long-term care facility, influenza vaccination in 2019–2020, number of nights hospitalized between 2015 and 2019 and presence of predisposing comorbidities or medical therapies. Estimates marked by an asterisk are statistically significantly different from the corresponding estimates for 70–79-year-olds.

	70-79	80-89	90-115
Comirnaty 0-20	41	44	46
Comirnaty 21-83	67	47*	62
Comirnaty 84+	66	66	31
Comirnaty + Comirnaty 0-13	87	66	100
Comirnaty + Comirnaty 14-90	95	87*	89
Comirnaty + Comirnaty 91-180	87	81*	88
Comirnaty + Comirnaty 181+	73	65	48*
Comirnaty + Comirnaty + Comirnaty 0-13	91	90	67*
Comirnaty + Comirnaty + Comirnaty 14-60	96	94	91
Comirnaty + Comirnaty + Comirnaty 61+	91	88	79*
Comirnaty + Comirnaty + Spikevax 0-13	92	69*	100
Comirnaty + Comirnaty + Spikevax 14-60	94	87	74
Comirnaty + Comirnaty + Spikevax 61+	89	81	24*
Spikevax 0-20	58	100	2
Spikevax 21-83	76	83	-61*
Spikevax 84+	89	35	53
Spikevax + Spikevax 0-13	100	61	100
Spikevax + Spikevax 14-90	98	81	100
Spikevax + Spikevax 91-180	87	74	70
Spikevax + Spikevax 181+	79	68	57
Spikevax + Spikevax + Comirnaty 0-13	100	78	-187
Spikevax + Spikevax + Comirnaty 14-60	93	88	100
Spikevax + Spikevax + Comirnaty 61+	94	82	100
Spikevax + Spikevax + Spikevax 0-13	91	93	62
Spikevax + Spikevax + Spikevax 14-60	99	95	86*
Spikevax + Spikevax + Spikevax 61+	89	87	73
Vaxzevria 0-20	28	100	100
Vaxzevria 21-83	68	100	100
Vaxzevria 84+	52	-29	100
Vaxzevria + Vaxzevria 0-13	100	100	100
Vaxzevria + Vaxzevria 14-90	87	22	100
Vaxzevria + Vaxzevria 91-180	75	100	100
Vaxzevria + Vaxzevria 181+	50	68	100
Vaxzevria + Vaxzevria + Comirnaty 0-13	85	100	100
Vaxzevria + Vaxzevria + Comirnaty 14-60	98	100	100
Vaxzevria + Vaxzevria + Comirnaty 61+	89	76	100
Vaxzevria + Vaxzevria + Spikevax 0-13	92	100	100
Vaxzevria + Vaxzevria + Spikevax 14-60	95	100	100
Vaxzevria + Vaxzevria + Spikevax 61+	88	100	100

Table S11: VE against Covid-19-related hospital admission by presence of comorbidities Vaccine effectiveness (in %) quantified as one minus the hazard ratio stratified by presence of predisposing comorbidities or medical therapies and adjusted for age, sex, region of residence, residence in a long-term care facility, influenza vaccination in 2019–2020 and number of nights hospitalized between 2015 and 2019. Estimates marked by an asterisk are statistically significantly different from the corresponding estimates for individuals without predisposing comorbidities or medical therapies.

	None	Moderate	High
Comirnaty 0-20	56	43	41
Comirnaty 21-83	54	82*	53
Comirnaty 84+	57	64	64
Comirnaty + Comirnaty 0-13	88	100	67
Comirnaty + Comirnaty 14-90	96	90	92
Comirnaty + Comirnaty 91-180	92	83*	82*
Comirnaty + Comirnaty 181+	80	68	63*
Comirnaty + Comirnaty + Comirnaty 0-13	96	89	86*
Comirnaty + Comirnaty + Comirnaty 14-60	98	95*	93*
Comirnaty + Comirnaty + Comirnaty 61+	95	87*	87*
Comirnaty + Comirnaty + Spikevax 0-13	94	94	80
Comirnaty + Comirnaty + Spikevax 14-60	97	93	88*
Comirnaty + Comirnaty + Spikevax 61+	98	78*	82*
Spikevax 0-20	54	100	64
Spikevax 21-83	80	87	58
Spikevax 84+	100	62	52
Spikevax + Spikevax 0-13	100	100	75
Spikevax + Spikevax 14-90	91	87	95
Spikevax + Spikevax 91-180	97	77	78
Spikevax + Spikevax 181+	85	66	71
Spikevax + Spikevax + Comirnaty 0-13	100	68	74
Spikevax + Spikevax + Comirnaty 14-60	92	88	92
Spikevax + Spikevax + Comirnaty 61+	100	100	82
Spikevax + Spikevax + Spikevax 0-13	100	91	85
Spikevax + Spikevax + Spikevax 14-60	100	98	95
Spikevax + Spikevax + Spikevax 61+	94	85	85
Vaxzevria 0-20	1	100	17
Vaxzevria 21-83	100	-4	80
Vaxzevria 84+	-110	-1	100
Vaxzevria + Vaxzevria 0-13	100	100	100
Vaxzevria + Vaxzevria 14-90	100	100	65
Vaxzevria + Vaxzevria 91-180	81	95	61
Vaxzevria + Vaxzevria 181+	88	73	19
Vaxzevria + Vaxzevria + Comirnaty 0-13	100	100	70
Vaxzevria + Vaxzevria + Comirnaty 14-60	100	90	100
Vaxzevria + Vaxzevria + Comirnaty 61+	100	95	79
Vaxzevria + Vaxzevria + Spikevax 0-13	67	100	100
Vaxzevria + Vaxzevria + Spikevax 14-60	93	91	96
Vaxzevria + Vaxzevria + Spikevax 61+	80	88	90

Table S12: VE against Covid-19-related hospital admission in 2021 Q4, i.e., between October 1 and December 31. Vaccine effectiveness (in %) quantified as one minus the hazard ratio adjusted for age, sex, region of residence, residence in a long-term care facility, influenza vaccination in 2019–2020, number of nights hospitalized between 2015 and 2019 and presence of predisposing comorbidities or medical therapies.

	Cases	P-years	MLE	LCI	UCI	p-value[1]
Not vaccinated	150	11015	.	.	.	.
Comirnaty 0-20	<5	411	65	-40	91	.
Comirnaty 21-83	5	773	53	-16	81	.
Comirnaty 84+	15	3312	68	45	81	.
Comirnaty + Comirnaty 0-13	<5	754	83	31	96	.
Comirnaty + Comirnaty 14-90	6	4694	90	78	96	.
Comirnaty + Comirnaty 91-180	157	103650	89	85	91	.
Comirnaty + Comirnaty 181+	76	19396	79	71	84	.
Comirnaty + Comirnaty + Comirnaty 0-13	23	16526	93	88	95	.
Comirnaty + Comirnaty + Comirnaty 14-60	18	18309	96	93	97	.
Comirnaty + Comirnaty + Comirnaty 61+	<5	1308	94	75	99	.
Comirnaty + Comirnaty + Spikevax 0-13	0	808	100	.	.	<0.001
Comirnaty + Comirnaty + Spikevax 14-60	<5	354	75	-3	94	.
Comirnaty + Comirnaty + Spikevax 61+	0	100	100	.	.	0.021
Spikevax 0-20	0	73	100	.	.	0.141
Spikevax 21-83	<5	196	60	-189	94	.
Spikevax 84+	<5	621	91	35	99	.
Spikevax + Spikevax 0-13	0	129	100	.	.	0.038
Spikevax + Spikevax 14-90	<5	862	92	42	99	.
Spikevax + Spikevax 91-180	22	12397	88	81	93	.
Spikevax + Spikevax 181+	6	2085	87	70	94	.
Spikevax + Spikevax + Comirnaty 0-13	<5	481	79	13	95	.
Spikevax + Spikevax + Comirnaty 14-60	<5	458	82	28	96	.
Spikevax + Spikevax + Comirnaty 61+	0	15	100	.	.	0.304
Spikevax + Spikevax + Spikevax 0-13	<5	1288	93	73	98	.
Spikevax + Spikevax + Spikevax 14-60	0	1148	100	.	.	<0.001
Spikevax + Spikevax + Spikevax 61+	0	29	100	.	.	0.145
Vaxzevria 0-20	0	<5	100	.	.	0.953
Vaxzevria 21-83	0	<5	100	.	.	0.807
Vaxzevria 84+	<5	192	62	-171	95	.
Vaxzevria + Vaxzevria 0-13	0	<5	100	.	.	0.894
Vaxzevria + Vaxzevria 14-90	0	295	100	.	.	0.023
Vaxzevria + Vaxzevria 91-180	21	8040	79	67	87	.
Vaxzevria + Vaxzevria 181+	6	411	23	-76	67	.
Vaxzevria + Vaxzevria + Comirnaty 0-13	<5	528	90	26	99	.
Vaxzevria + Vaxzevria + Comirnaty 14-60	<5	283	84	-16	98	.
Vaxzevria + Vaxzevria + Comirnaty 61+	0	14	100	.	.	0.365
Vaxzevria + Vaxzevria + Spikevax 0-13	0	156	100	.	.	0.012
Vaxzevria + Vaxzevria + Spikevax 14-60	0	42	100	.	.	0.171
Vaxzevria + Vaxzevria + Spikevax 61+	0	<5	100	.	.	0.695

MLE, maximum likelihood estimate;

LCI/UCI, lower/upper limit of the 95% Wald confidence interval

[1] Likelihood-ratio test

Table S13: VE against Covid-19-related hospital admission in 2022 Q1, i.e., between January 1 and March 31. Vaccine effectiveness (in %) quantified as one minus the hazard ratio adjusted for age, sex, region of residence, residence in a long-term care facility, influenza vaccination in 2019–2020, number of nights hospitalized between 2015 and 2019 and presence of predisposing comorbidities or medical therapies.

	Cases	P-years	MLE	LCI	UCI	p-value[1]
Not vaccinated	292	8929	.	.	.	.
Comirnaty 0-20	<5	122	24	-137	76	.
Comirnaty 21-83	12	460	22	-38	56	.
Comirnaty 84+	15	1073	61	34	77	.
Comirnaty + Comirnaty 0-13	<5	200	67	-31	92	.
Comirnaty + Comirnaty 14-90	9	2993	91	83	95	.
Comirnaty + Comirnaty 91-180	28	3323	75	62	83	.
Comirnaty + Comirnaty 181+	124	9161	58	48	66	.
Comirnaty + Comirnaty + Comirnaty 0-13	17	4384	84	73	90	.
Comirnaty + Comirnaty + Comirnaty 14-60	89	51159	94	92	95	.
Comirnaty + Comirnaty + Comirnaty 61+	357	72954	87	85	89	.
Comirnaty + Comirnaty + Spikevax 0-13	10	1963	82	66	91	.
Comirnaty + Comirnaty + Spikevax 14-60	26	8868	91	87	94	.
Comirnaty + Comirnaty + Spikevax 61+	39	5501	83	76	88	.
Spikevax 0-20	<5	42	38	-340	91	.
Spikevax 21-83	<5	122	81	-33	97	.
Spikevax 84+	7	204	23	-63	64	.
Spikevax + Spikevax 0-13	0	40	100	.	.	0.082
Spikevax + Spikevax 14-90	<5	485	79	43	92	.
Spikevax + Spikevax 91-180	5	594	74	36	89	.
Spikevax + Spikevax 181+	17	1214	60	34	75	.
Spikevax + Spikevax + Comirnaty 0-13	<5	185	81	-36	97	.
Spikevax + Spikevax + Comirnaty 14-60	<5	1709	92	80	97	.
Spikevax + Spikevax + Comirnaty 61+	10	2076	88	77	94	.
Spikevax + Spikevax + Spikevax 0-13	<5	716	84	51	95	.
Spikevax + Spikevax + Spikevax 14-60	8	5482	96	91	98	.
Spikevax + Spikevax + Spikevax 61+	41	5919	85	78	89	.
Vaxzevria 21-83	0	<5	100	.	.	0.894
Vaxzevria 84+	0	56	100	.	.	0.058
Vaxzevria + Vaxzevria 14-90	0	<5	100	.	.	0.869
Vaxzevria + Vaxzevria 91-180	<5	142	37	-154	85	.
Vaxzevria + Vaxzevria 181+	12	872	51	12	73	.
Vaxzevria + Vaxzevria + Comirnaty 0-13	<5	390	78	11	95	.
Vaxzevria + Vaxzevria + Comirnaty 14-60	<5	2761	99	91	100	.
Vaxzevria + Vaxzevria + Comirnaty 61+	12	2420	86	75	92	.
Vaxzevria + Vaxzevria + Spikevax 0-13	<5	317	89	19	98	.
Vaxzevria + Vaxzevria + Spikevax 14-60	<5	1531	94	81	98	.
Vaxzevria + Vaxzevria + Spikevax 61+	5	936	87	68	95	.

MLE, maximum likelihood estimate;

LCI/UCI, lower/upper limit of the 95% Wald confidence interval

[1] Likelihood-ratio test

Table S14: Negative control outcome analysis Ratio comparing the hazard of injury, poisoning and certain other consequences of external causes in vaccinated study subjects with the corresponding hazard in the unvaccinated, adjusted for age, sex, region of residence, residence in a long-term care facility, influenza vaccination in 2019–2020, number of nights hospitalized between 2015 and 2019 and presence of predisposing comorbidities or medical therapies. Estimates marked by an asterisk are statistically significantly different from one.

	Cases	P-years	MLE	LCI	UCI	p-value[1]	
Not vaccinated	7980	243895	.	.	.	.	
Comirnaty 0-20	1300	40514	0.92	0.86	0.98	.	*
Comirnaty 21-83	3704	113710	0.92	0.87	0.97	.	*
Comirnaty 84+	508	9975	1.02	0.92	1.12	.	
Comirnaty + Comirnaty 0-13	933	26176	0.95	0.88	1.02	.	
Comirnaty + Comirnaty 14-90	5129	142175	0.95	0.91	1.00	.	
Comirnaty + Comirnaty 91-180	5145	154744	0.95	0.90	1.01	.	
Comirnaty + Comirnaty 181+	1385	31053	1.07	1.00	1.16	.	
Comirnaty + Comirnaty + Comirnaty 0-13	622	20430	0.94	0.85	1.03	.	
Comirnaty + Comirnaty + Comirnaty 14-60	2067	67674	0.97	0.90	1.04	.	
Comirnaty + Comirnaty + Comirnaty 61+	2593	72609	0.94	0.87	1.02	.	
Comirnaty + Comirnaty + Spikevax 0-13	78	2708	1.00	0.79	1.26	.	
Comirnaty + Comirnaty + Spikevax 14-60	261	8958	0.97	0.84	1.11	.	
Comirnaty + Comirnaty + Spikevax 61+	220	5434	1.14	0.98	1.33	.	
Spikevax 0-20	223	4853	1.18	1.03	1.35	.	*
Spikevax 21-83	620	13710	1.14	1.04	1.24	.	*
Spikevax 84+	119	1595	1.48	1.23	1.78	.	*
Spikevax + Spikevax 0-13	159	2997	1.27	1.08	1.49	.	*
Spikevax + Spikevax 14-90	861	16195	1.28	1.18	1.38	.	*
Spikevax + Spikevax 91-180	797	17227	1.22	1.12	1.33	.	*
Spikevax + Spikevax 181+	172	3209	1.26	1.08	1.48	.	*
Spikevax + Spikevax + Comirnaty 0-13	26	639	1.16	0.78	1.71	.	
Spikevax + Spikevax + Comirnaty 14-60	87	2077	1.22	0.98	1.52	.	
Spikevax + Spikevax + Comirnaty 61+	82	2014	1.02	0.82	1.29	.	
Spikevax + Spikevax + Spikevax 0-13	83	1932	1.21	0.97	1.51	.	
Spikevax + Spikevax + Spikevax 14-60	270	6380	1.21	1.06	1.39	.	*
Spikevax + Spikevax + Spikevax 61+	300	5714	1.24	1.09	1.42	.	*
Vaxzevria 0-20	57	2722	0.81	0.62	1.05	.	
Vaxzevria 21-83	227	7894	1.03	0.89	1.18	.	
Vaxzevria 84+	21	469	1.48	0.96	2.27	.	
Vaxzevria + Vaxzevria 0-13	36	1487	0.83	0.60	1.16	.	
Vaxzevria + Vaxzevria 14-90	201	8135	0.88	0.76	1.02	.	
Vaxzevria + Vaxzevria 91-180	222	8915	0.99	0.86	1.14	.	
Vaxzevria + Vaxzevria 181+	40	1328	1.18	0.86	1.62	.	
Vaxzevria + Vaxzevria + Comirnaty 0-13	21	901	0.98	0.64	1.51	.	
Vaxzevria + Vaxzevria + Comirnaty 14-60	66	2985	0.94	0.73	1.20	.	
Vaxzevria + Vaxzevria + Comirnaty 61+	52	2393	0.79	0.60	1.05	.	
Vaxzevria + Vaxzevria + Spikevax 0-13	14	463	1.22	0.72	2.07	.	
Vaxzevria + Vaxzevria + Spikevax 14-60	42	1537	1.06	0.78	1.45	.	
Vaxzevria + Vaxzevria + Spikevax 61+	24	918	0.89	0.59	1.34	.	

MLE, maximum likelihood estimate; LCI/UCI, lower/upper limit of the 95% Wald confidence interval

[1] Likelihood-ratio test

Figure S1: Distribution of SARS-CoV-2 variants by calendar month A subset of SARS-CoV-2-positive specimens are regularly selected for sequencing to assess the distribution of SARS-CoV-2 variants in Finland. The results are recorded in the National Infectious Diseases Register. The figure shows the relative frequency of each variant between December 27, 2020, and March 31, 2022 in the subpopulation aged 70 years and over. The corresponding number of sequenced specimens as well as the percentage of specimens that were sequenced are given below the x-axis.

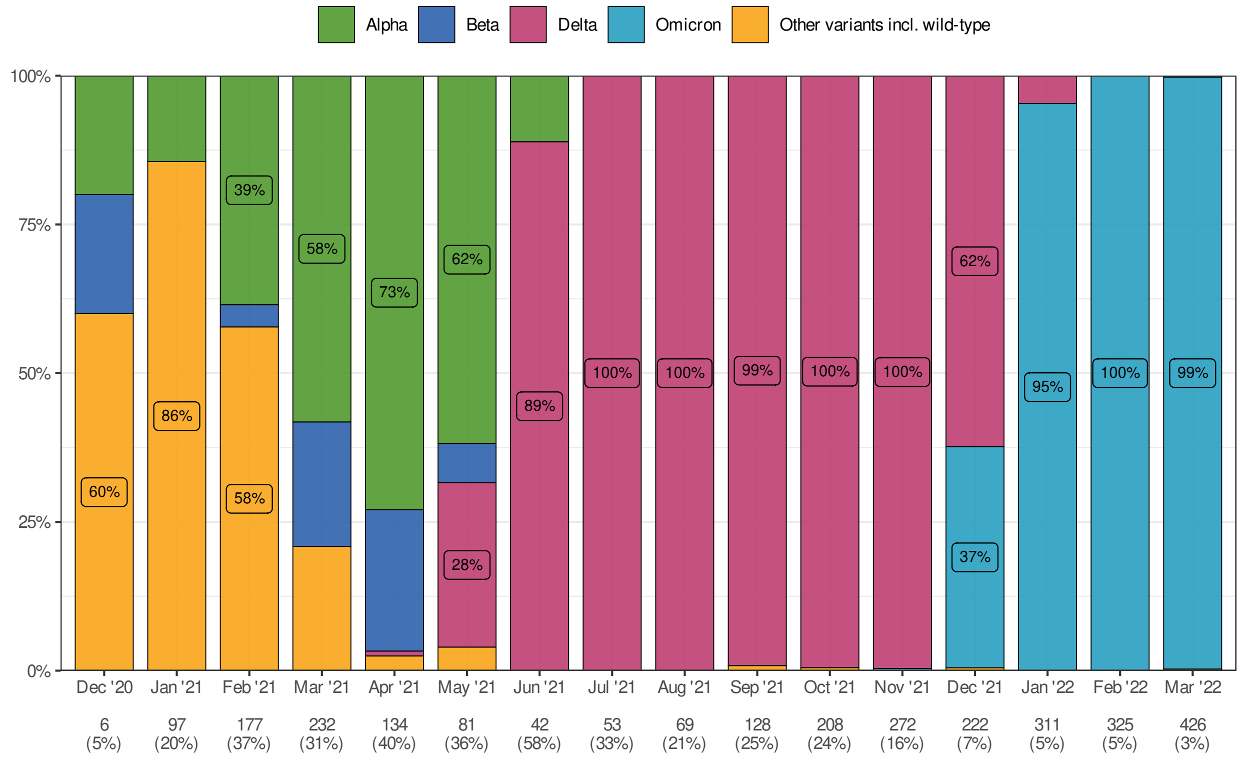

The Omicron subtypes BA.1, BA.2, XJ accounted for 69%, 27%, 4%, respectively, of all Omicron infections.

Figure S2: Distribution of exposure states by calendar month Relative distribution of person-years in the study of vaccine effectiveness against Covid-19 hospitalization from December 27, 2020, to March 31, 2022.

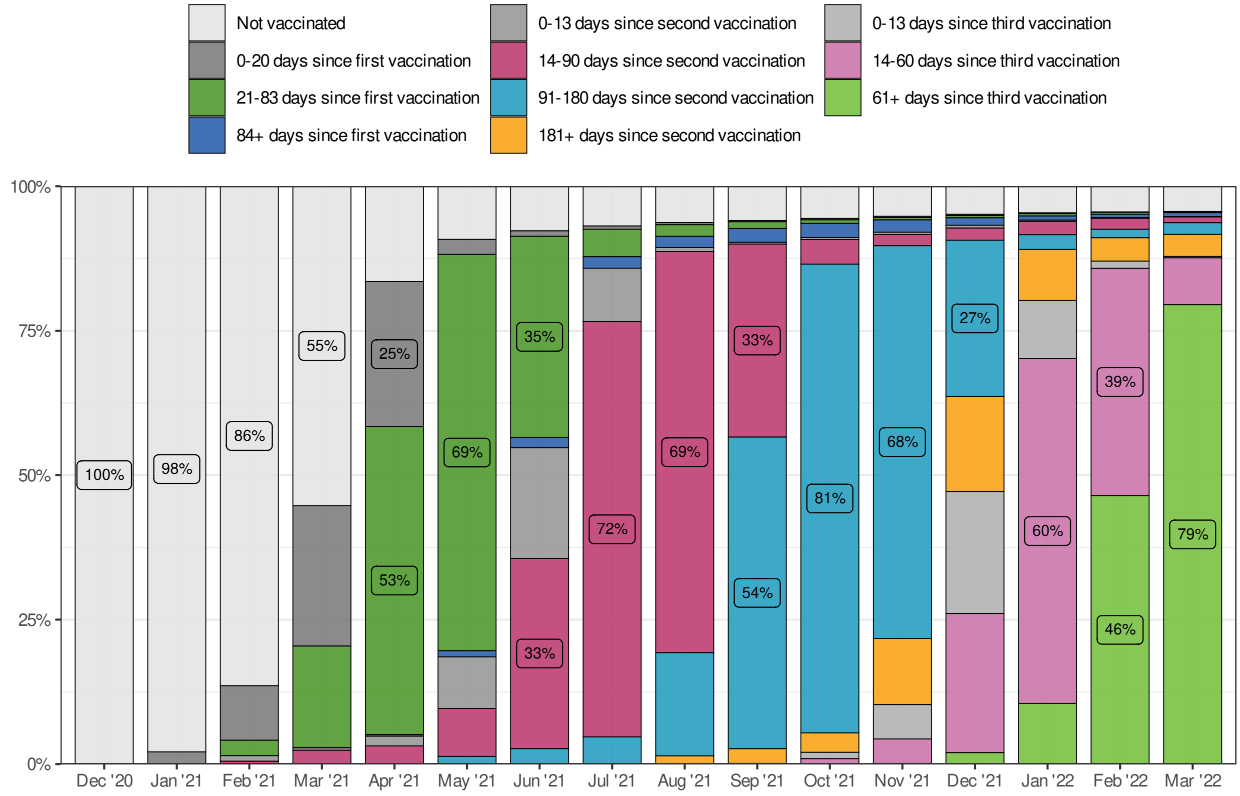

Figure S3: Number of cases by calendar month Number of Covid-19-related hospital admissions and Covid-19-related ICU admissions in the study, i.e., between December 27, 2020, and March 31, 2022.

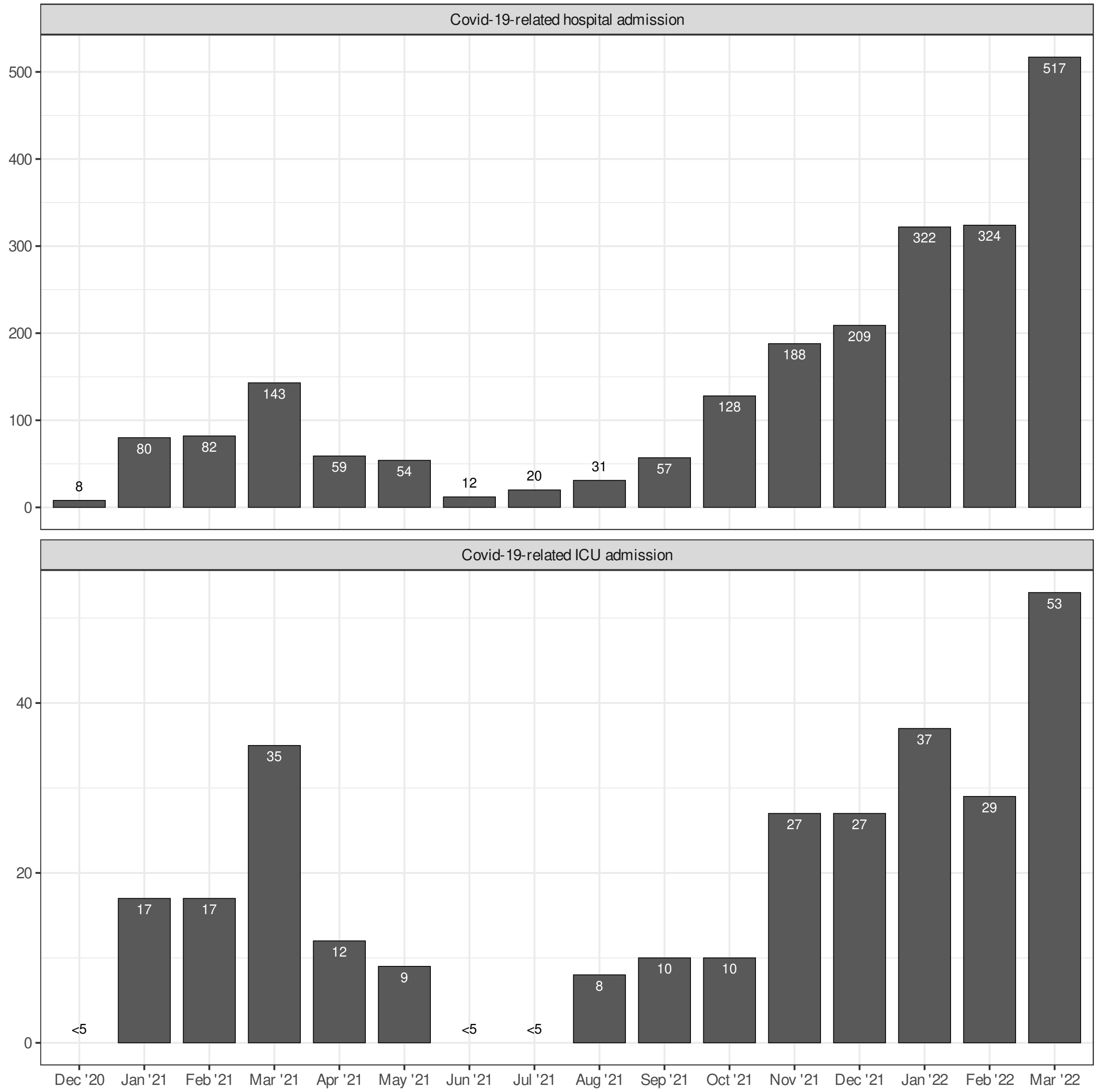

Figure S4: Cumulative hazard of Covid-19-related hospital admission between December 27, 2020, to March 31, 2022 by calendar time in days and by vaccine, dose and days since last vaccination. For clarity, only the graphs for unvaccinated study subjects and study subjects vaccinated with Comirnaty are shown. The graphs are based on the predicted survival curves adjusted for age, sex, region of residence, residence in a long-term care facility, influenza vaccination in 2019–2020, number of nights hospitalized between 2015 and 2019 and presence of predisposing comorbidities or medical therapies.

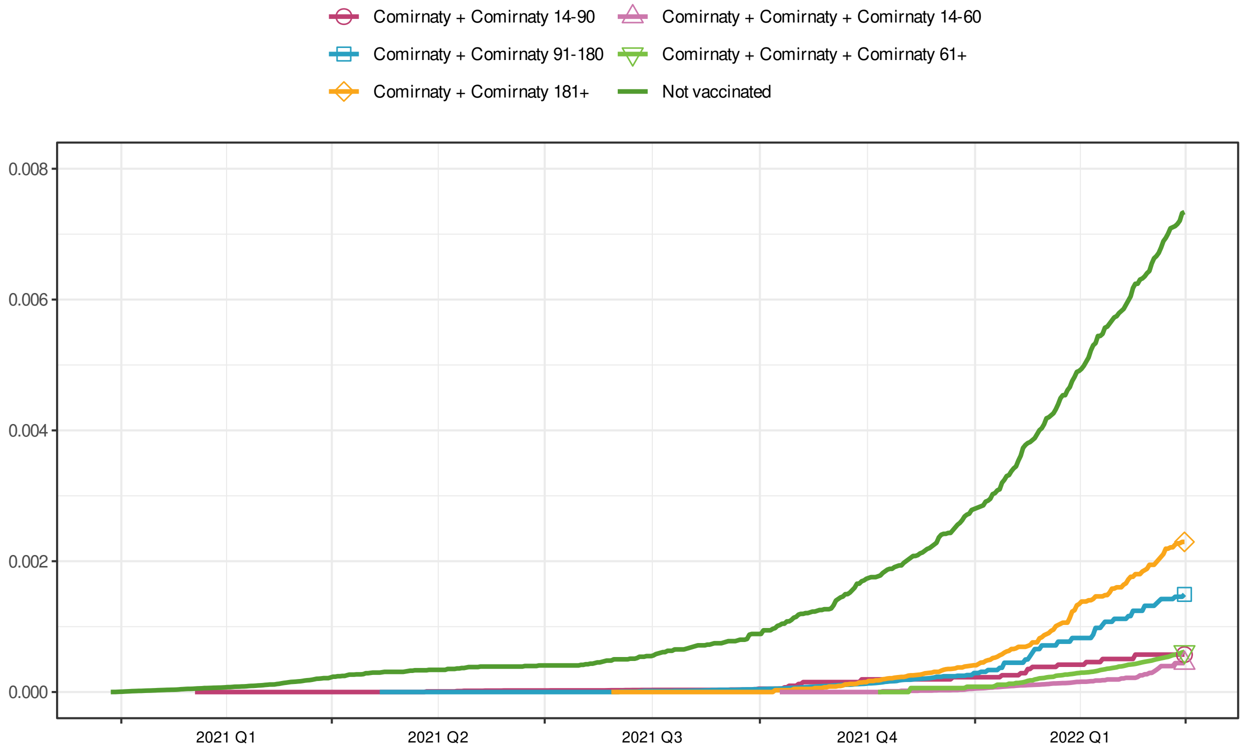

Figure S5: Cumulative hazard of negative control outcome Cumulative hazard of injury, poisoning and certain other consequences of external causes between December 27, 2020, to March 31, 2022 by calendar time in days and by vaccine, dose and days since last vaccination. For clarity, only the graphs for unvaccinated study subjects and study subjects vaccinated with Comirnaty are shown. The graphs are based on the predicted survival curves adjusted for age, sex, region of residence, residence in a long-term care facility, influenza vaccination in 2019–2020, number of nights hospitalized between 2015 and 2019 and presence of predisposing comorbidities or medical therapies.

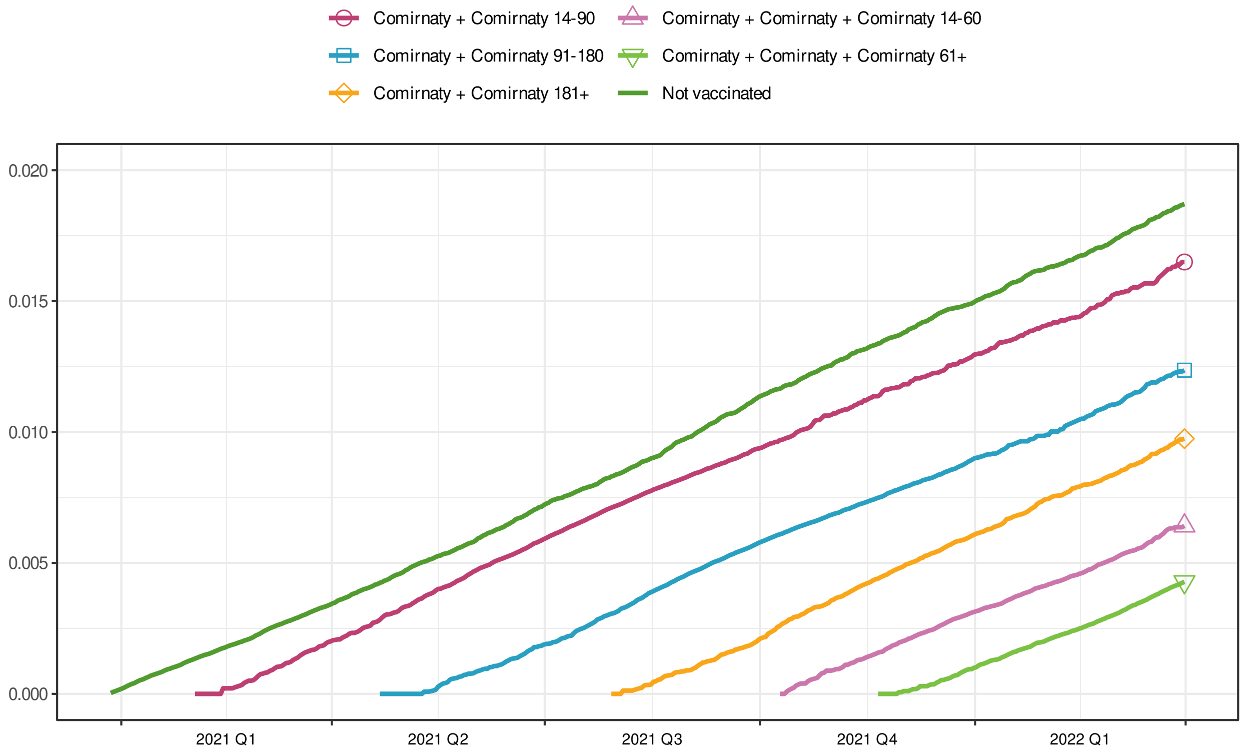

Supplement: Supplementary file 1 — Additional file 1: Table S1. Highly predisposing comorbidities or medical therapies. Table S2. Moderately predisposing comorbidities or medical therapies. Table S3. Enrollment of the study cohort. Table S4. Distribution of baseline characteristics. Table S5. Number of days until first dose. Table S6. Number of COVID-19-related hospital admissions by vaccine, dose and days since last vaccination. Table S7. Number of COVID-19-related ICU admissions by vaccine, dose and days since last vaccination. Table S8. VE against COVID-19-related hospital admission. Table S9. VE against COVID-19-related ICU admission. Table S10. VE against COVID-19-related hospital admission by age group. Table S11. VE against COVID-19-related hospital admission by presence of comorbidities. Table S12. VE against COVID-19-related hospital admission in 2021 Q4, i.e., between October 1 and December 31. Table S13. VE against COVID-19-related hospital admission in 2022 Q1, i.e., between January 1 and March 31. Table S14. Negative control outcome analysis. Figure S1. Distribution of SARS-CoV-2 variants by calendar month. Figure S2. Distribution of exposure states by calendar month. Figure S3. Number of cases by calendar month. Figure S4. Cumulative hazard of COVID-19-related hospital admission between December 27, 2020, to March 31, 2022 by calendar time in days and by vaccine, dose and days since last vaccination. Figure S5. Cumulative hazard of negative control outcome. [file 12879_2022_7814_MOESM1_ESM.pdf]
